# Transcriptional Regulation of Human and Rat Hepatic Lipid Metabolism by the Grapefruit Flavonoid Naringenin: Role of PPARα, PPARγ and LXRα

**DOI:** 10.1371/journal.pone.0012399

**Published:** 2010-08-25

**Authors:** Jonathan Goldwasser, Pazit Y. Cohen, Eric Yang, Patrick Balaguer, Martin L. Yarmush, Yaakov Nahmias

**Affiliations:** 1 Center for Engineering in Medicine, Shriners Burns Hospital, Boston, Massachusetts, United States of America; 2 Harvard-MIT Division of Health Science and Technology, Cambridge, Massachusetts, United States of America; 3 The Selim and Rachel Benin School of Engineering, The Hebrew University of Jerusalem, Jerusalem, Israel; 4 Massachusetts General Hospital, Harvard Medical School, Boston, Massachusetts, United States of America; 5 INSERM, Univ Montpellier I, Montpellier, France; National Institute on Aging, United States of America

## Abstract

Disruption of lipid and carbohydrate homeostasis is an important factor in the development of prevalent metabolic diseases such as diabetes, obesity, and atherosclerosis. Therefore, small molecules that could reduce insulin dependence and regulate dyslipidemia could have a dramatic effect on public health. The grapefruit flavonoid naringenin has been shown to normalize lipids in diabetes and hypercholesterolemia, as well as inhibit the production of HCV. Here, we demonstrate that naringenin regulates the activity of nuclear receptors PPARα, PPARγ, and LXRα. We show it activates the ligand-binding domain of both PPARα and PPARγ, while inhibiting LXRα in GAL4-fusion reporters. Using TR-FRET, we show that naringenin is a partial agonist of LXRα, inhibiting its association with Trap220 co-activator in the presence of TO901317. In addition, naringenin induces the expression of PPARα co-activator, PGC1α. The flavonoid activates PPAR response element (PPRE) while suppressing LXRα response element (LXRE) in human hepatocytes, translating into the induction of PPAR-regulated fatty acid oxidation genes such as CYP4A11, ACOX, UCP1 and ApoAI, and inhibition of LXRα-regulated lipogenesis genes, such as FAS, ABCA1, ABCG1, and HMGR. This effect results in the induction of a *fasted*-like state in primary rat hepatocytes in which fatty acid oxidation increases, while cholesterol and bile acid production decreases. Our findings explain the myriad effects of naringenin and support its continued clinical development. Of note, this is the first description of a non-toxic, naturally occurring LXRα inhibitor.

## Introduction

The liver is the hub of lipid and carbohydrate homeostasis [1]. Dysregulation of this homeostasis has been implicated in disease processes, such as atherogenesis, insulin resistance, and hypermetabolism [2], [3]. Metabolic conditions, such as insulin resistance, may be partly attributable to ‘western-style diets’ and are associated with medical expenditures and lost productivity totaling over $130 billion annually [4]. Therefore, drugs or dietary supplements that could potentially reduce insulin dependence and regulate dyslipidemia could have a dramatic effect on healthcare expenditures and public health.

One group of compounds previously shown to have hypolipidemic and anti-inflammatory properties both *in vivo* and *in vitro* are citrus flavonoids [5], [6]. The abundant flavonoid aglycone naringenin, which is responsible for the bitter taste in grapefruits, has been extensively studied in recent years. In vivo studies have demonstrated its potential as a normolipidemic agent: in a recent clinical trial, naringenin was shown to reduce circulating levels of low-density lipoprotein (LDL) by 17% in hypercholesterolemic patients [7]. Similarly, the cholesterol-lowering effects of naringenin have been demonstrated in rabbits [8], [9] and rats [10]. In HepG2 cells, naringenin was shown to reduce the secretion of VLDL [11], [12] through the inhibition of ACAT2 [11] and MTP [13], [14], enzymes critical for VLDL assembly. Naringenin was also shown to induce LDL-R transcription through PI3K activation upstream of SREBP-1a [11], [14]. Other studies demonstrated that naringenin inhibited HMG CoA reductase (HMGR), while activating enzymes important in fatty acid oxidation such as CYP4A1 [15]. Naringenin's myriad effects suggest that the flavonoid may be targeting transcriptional regulation of metabolism through nuclear receptors (NRs), a family of ligand-activated transcription factors, which play a critical role in the regulation of lipid metabolism. Strengthening this hypothesis is the anecdotal report that naringenin binds to LXRα [14] and more recently, that the flavonoid induces PPRE activity in U-2OS cells [16].

In this study, we demonstrate that naringenin is an agonist of PPARα and PPARγ, and a partial agonist of LXRα. We show that naringenin induces the activation of PPARα and PPARγ ligand-binding domain (LBD) in GAL4-fusion protein reporters and induces PPRE activity in Huh7.5 human hepatoma cells. Using an *in vitro* TR-FRET assay we demonstrate that this interaction does not change the binding of PGC1α co-activator peptide to recombinant PPARα ligand binding domain.

Concomitantly, naringenin inhibits the activation of the LXRα LBD in a GAL4-fusion protein reporter in the presence of the LXRα agonist TO901317. Using an *in vitro* TR-FRET assay, we demonstrate that this effect is mediated by the inhibition of the binding of the Trap220/Drip-2 co-activator peptide to recombinant LXRα LBD. Expectedly, naringenin also inhibits LXRE activity in Huh7.5 cells. We show that the induction of PPARα and inhibition of LXRα induces the expected transcriptional changes in hepaotcytes, upregulating genes important in fatty acid oxidation and down-regulating cholesterol and fatty acid synthesis. These effects result in the induction of a fasted-like state in primary hepatocytes, in which production of triglycerides and bile acids is inhibited and ketone body generation increases.

## Results

### Naringenin activates PPARα and PPARγ

**Figure 1:**
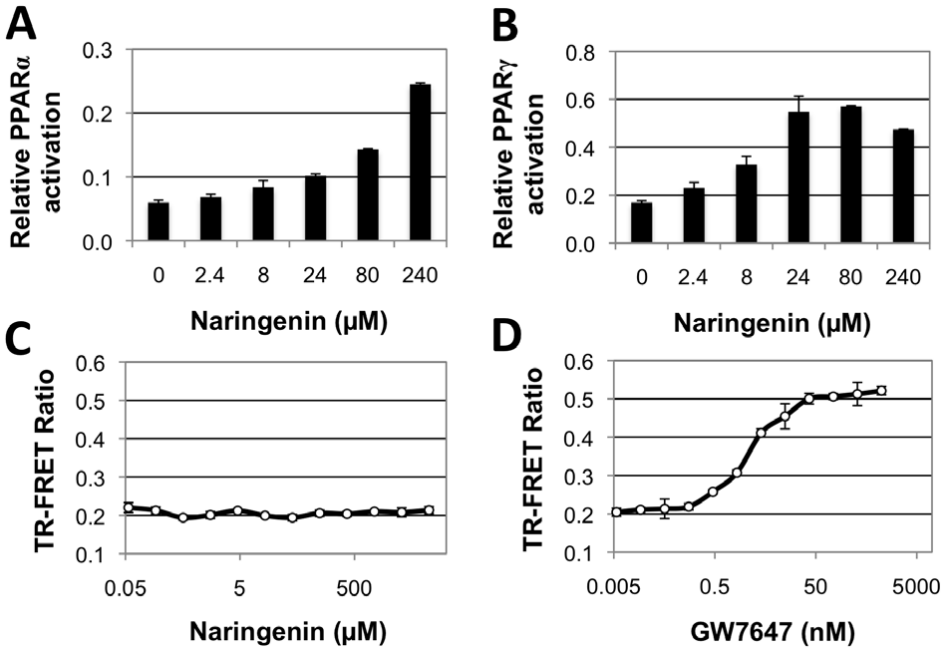
Naringenin induces activation of PPARα and PPARγ ligand-binding domains. HG_5_LN reporter cells expressing GAL4-PPARα (a) and GAL4-PPARγ (b) reporters were treated with increasing concentrations of naringenin. Naringenin dose-dependently activated PPARα reaching 24%±0.2% induction at 240 µM (P<0.001); and activated PPARγ up to 57%±0.3% at 80 µM (P<0.005). Data is presented as percent activation relative to 1 µM of classical agonists GW7647 and BRL49653, respectively. (c) LanthaScreen TR-FRET assay, demonstrating that naringenin did not affect the binding of the PGC1α co-activator peptide to recombinant PPARα LBD. (d) In contrast, the classical PPARα agonist GW7647 induces a dose-dependent binding of PGC1α to PPARα in the same assay.

The manifold effects of naringenin, include the induction of β-oxidation [17] and anti-inflammation [5], suggest an underlying mechanism, similar to the activities of PPARα and PPARγ agonists such as fibrates or thiazolidinediones (TZDs) [18], [19]. Therefore, naringenin activation of PPARα and PPARγ were investigated using the previously described HeLa reporter cell lines, HG_5_LN GAL4-PPARα and HG_5_LN GAL4-PPARγ [20]. In these cells, the PPAR LBD is fused to the GAL4 DNA binding domain and expressed constitutively. Upon binding to an agonist, the PPAR-GAL4 fusion protein activates a luciferase reporter [20]. Naringenin dose-dependently activated PPARα reaching 24%±0.2% induction at 240 µM (P<0.001) relative to 1 µM of the PPARα agonist GW7647 (**Fig. 1a**). Furthermore, naringenin activated PPARγ up to 57%±0.3% at 80 µM (P<0.005) relative to the PPARγ agonist 1 µM BRL49653 (**Fig. 1b**).

To further characterize the interaction between PPARα and naringenin, a LanthaScreen time-resolved fluorescence resonance energy transfer (TR-FRET) assay was performed. This cell-free system measures the ability of a compound to enhance the binding of a recombinant PPARα LBD to a PGC1α co-activator peptide, as measured by an increase in TR-FRET signal. While GW7647 showed a clear dose-dependent increase (EC_50_ = 2.5nM) in the binding of PGC1α to PPARα as expected (**Fig. 1d**), the binding of PGC1α to PPARα did not increase in the presence of naringenin (**Fig. 1c**), suggesting that naringenin's ability to activate PPARα does not directly involve enhancement of PPARα LBD binding to PGC1α.

One possibility is that naringenin induces the transcription of PGC1α itself, an effect that cannot be seen in the cell-free TR-FRET assay. Indeed, stimulation of Huh7 cells with 380 µM naringenin for 24 hours increased PGC1α mRNA abundance by 14-fold (p = 0.001) compared to DMSO-treated controls.

### Naringenin is a partial agonist of LXRα

**Figure 2:**
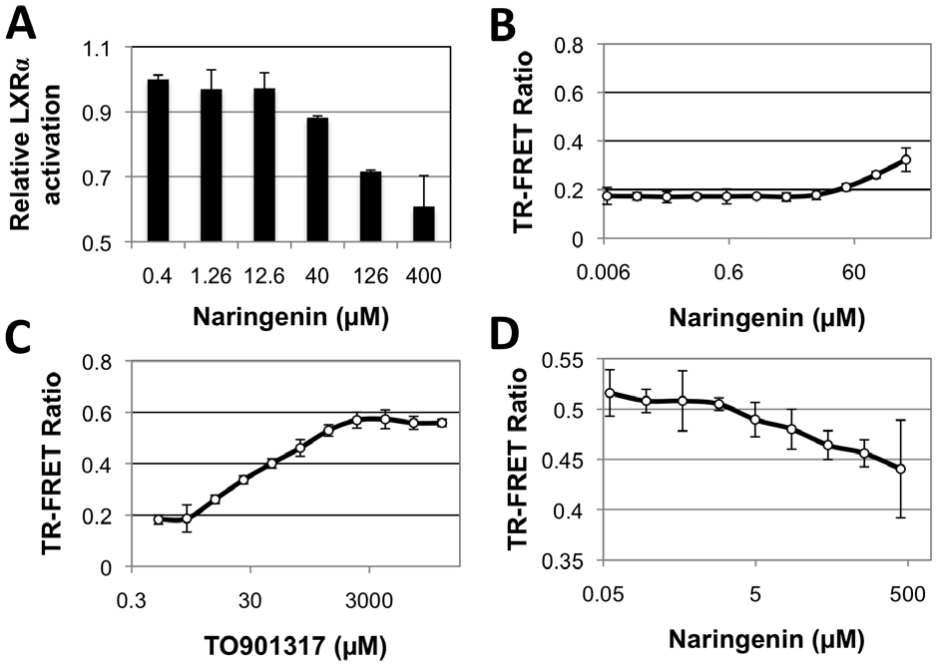
Naringenin is a partial agonist of LXRα ligand-binding domain. (a) LXR-alpha-UAS-bla HEK 293T cells were stimulated with 4.7 nM TO901317 and exposed to increasing concentrations of naringenin. Naringenin dose-dependently inhibited LXRα activity, reaching 28.4%±0.4% (p<0.01) and 39.1%±9.4% (p<0.05) at concentrations of 126 µM and 400 µM, respectively. (b-d) Lanthascreen TR-FRET assay, demonstrating that naringenin weakly increased the binding of Trap 220/Drip-2 co-activator peptide to recombinant LXRα LBD, and inhibited this binding in the presence of TO901317, LXRα classical agonist. (b) Naringenin is a weak agonist, enhancing the binding of the LXRα LBD to the Trap 220/Drip-2 co-activator moderately, yet significantly, in a dose-dependent manner reaching 38.0%±2.8% activation. (c) LXRα agonist TO901317 strongly enhanced co-activator binding. (d) When treated with 250 nM TO901317, increasing concentrations of naringenin led to an inhibition of the TR-FRET signal, reaching 15.0%±4.1% inhibition (p<0.01) at 133 µM.

Our group and others have shown that naringenin inhibits HMGR, an enzyme controlled by SREBP1c and in turn by the LXRα [21], [22]. In fact, there are some indications that naringenin binds LXRα *in vitro* [23]. To test naringenin's capacity to function as an LXRα antagonist, LXR-alpha-UAS-bla HEK 293T cells were stimulated with 4.7 nM TO901317 (corresponding to TO901217 EC_80_) and then treated with increasing concentrations of naringenin. Naringenin dose-dependently inhibited LXRα activity, reaching 28.4%±0.4% (p<0.01) and 39.1%±9.4% (p<0.05) at concentrations of 126 µM and 400 µM, respectively (**Fig. 2a**).

**Figure 3:**
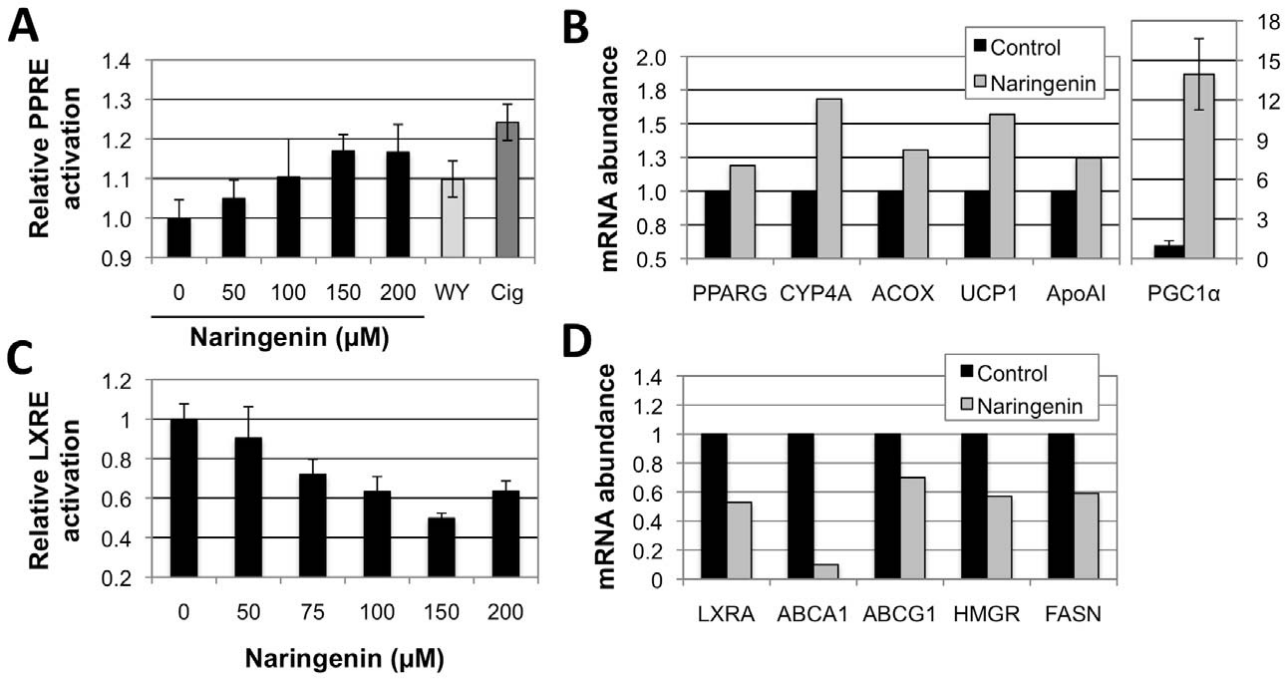
Naringenin activates PPRE-driven and inhibits LXRE-driven gene expression in human hepatocytes. (a) Naringenin dose-dependently enhanced PPRE activity, in Huh7 cells transiently transfected with a PPRE reporter, reaching 17%±7% (p<0.05) at 200 µM. Induction was not different from PPAR agonists WY14,643 and ciglitazone. (b) Naringenin induced the expression of PPARα coactivator PGC1α by 14-fold (p = 0.001) as well as PPARα-regulated fatty acid oxidation genes CYP4A11/22, ACOX, UCP1 and ApoAI. Huh7 cells were treated with naringenin for 24 hours and mRNA isolated and anlysed by qRT-PCR. (c) Naringenin dose-dependently suppressed LXRE activity, in Huh7 cells transiently transfected with a LXRE reporter, reaching a 50.3%±2.6% (p<0.001) inhibition at 150 µM. (d) Naringenin inhibited the expression of LXRα-regulated lipogenesis genes ABCA1, ABCG1, HMGR, and FASN. Cell viability under all conidtions was greater than 95%.

The interaction between LXRα and naringenin was further characterized using a Lanthascreen TR-FRET assay. Naringenin enhanced the binding of the LXRα LBD to the Trap 220/Drip-2 co-activator moderately, yet significantly, in a dose-dependent manner reaching 38.0%±2.8% activation (**Fig. 2b**) compared to the well-studied LXRα agonist, TO901317 (**Fig. 2c**). Notably, in the presence of 1 µM TO901317 (corresponding to TO901317 EC_80_), naringenin dose-dependently inhibited the binding of the Trap 220/Drip-2 co-activator to the LXRα LBD, reaching 11.6%±3% inhibition (p<0.01) at 133 µM (**Fig. 3d**). These results suggest that naringenin is a ligand and a partial agonist of LXRα.

### Naringenin induces PPRE and inhibits LXRE activity in hepatocytes

To explore the effect of naringenin on PPAR activation in hepatocytes, we quantified the activation of a PPAR response element (PPRE)-reporter in Huh7 cells. Naringenin treatment significantly and dose-dependently enhanced PPRE activity, reaching 17%±4% (p<0.01) at 150 µM (**Fig. 3a**). Similar levels of activation were observed when cells were exposed to the known PPAR agonists, WY14,643 (10%±5%) and ciglitazone (24%±5%). Notably, at 200 µM naringenin induction of PPRE was not significantly different then 10 µM WY14,643 (p = 0.25).

To test the ability of naringenin to inhibit LXRα activity in hepatocytes, we quantified the activation of LXR response element (LXRE)-reporter in Huh7 cells. Naringenin treatment significantly and dose-dependently decreased LXRE acitivty, reaching a 50.3%±2.6% inhibition at 150 µM (p<0.001; **Fig 3c**). By comparison, a recently published LXRα-specific antagonist, 5CPPSS-50 failed to inhibit LXRE activity under the same conditions (**Supp.** **Fig. 1**) and led to significant toxicity at higher doses.

### Naringenin-induced Gene and Metabolic changes in hepatocytes

To assess if PPARα activation by naringenin leads to induction of PPARα-regulated genes we stimulation Huh7 cells with 200 µM naringenin for 24 hours and quantified mRNA abuandance by qRT-PCR. Naringenin induced the expression of fatty acid oxidation genes CYP4A11, ACOX, UCP1 and ApoAI by 68%, 31%, 60%, and 25%, respectively (**Fig. 3b**). On the other hand, naringenin reduced the mRNA abuandance of LXRα-regulated genes ABCA1, ABCG1, HMGR, and FASN by 92%, 27%, 43%, and 41% respectively (**Fig. 3d** **)**. These results suggest a shift from lipogensis and cholesterol synthesis to lipolysis.

**Figure 4:**
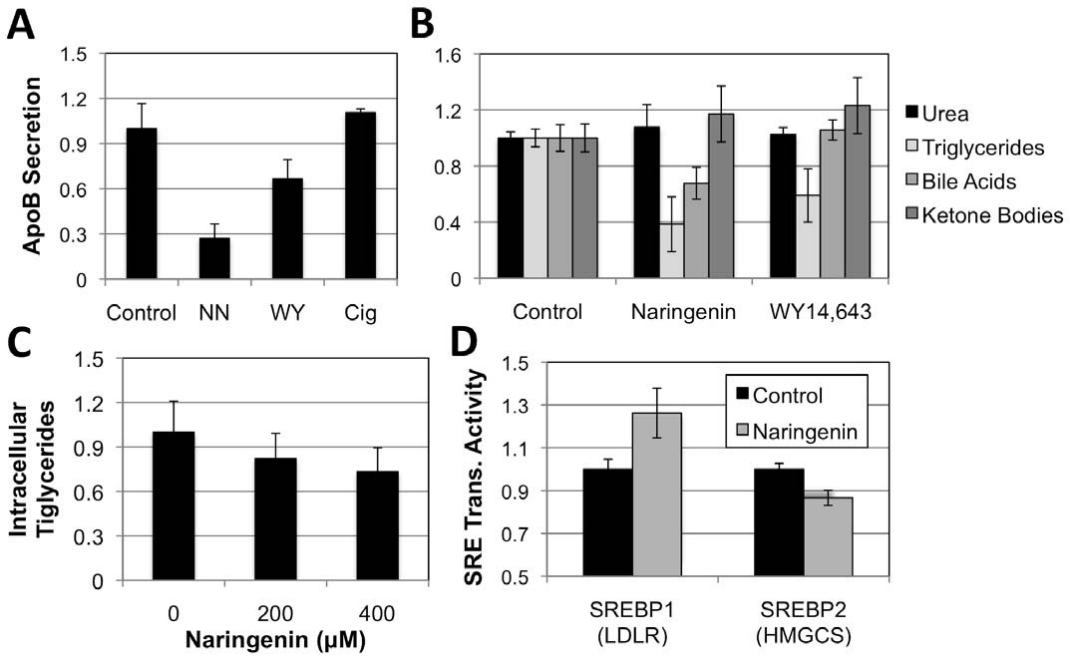
Naringenin induced a *fasted*-like state in hepatic lipid metabolism. (a) Huh7 cells were stimulated for 24 hours with 200 µM naringenin, 10 µM WY14,643, or 10 µM ciglitazone. Naringenin treatment led to a 73%±9% (p<0.001) reduction in ApoB production, while WY14,643 led to a 33%±12% (p<0.01) reduction. Treatment with cigilitazone did not lead to a significant change in VLDL production. (b) Primary rat hepatocytes were stimulated with 200 µM naringenin or 10 µM WY14,643. Naringenin treatment led to a 61% (p<0.001) reduction in triglyceride production and 17% increase in ketone body formation, not different from WY14,643. However, naringenin treatment led to a 32%±11% (p = 0.005) reduction in bile salt production, while WY14,643 did not. Urea accumulation in the media did not change significantly. (c) Intracellular levels of triglycerides in primary rat hepatocytes stimulated with naringenin. A slight decrease is observed. (d) Naringenin effect on SRE-driven gene expression. We show that naringein induces LDLR transcription by 26% (p = 0.02) while inhibiting HMGCS transcription by 13% (p = 0.001). It is thought that each promoter is regulated by a different SREBP isoform.

Interestingly, Huff and coworkers previously demonstrated that naringenin activated SREBP1a-dependent LDLR expresion [11], [14]. As SREBP is regulated by LXRα we studied the gene expression of SREBP1/2 regulated LDLR and HMGCS promoters in Huh7 cells using reporter constructs. We show that naringenin increases LDLR transcription by 26%±11%, but decreases HMGCS transcription by 13%±3% (**Fig 4d**). HMGCS is regulated by SREBP2 rather than SREBP1 and like HMGR plays a role in cholesterol synthesis.

ApoB100 is the structural protein of VLDL whose production is blocked by naringenin [21]. As our results suggest that naringenin acts through PPARα induction, we examined whether PPARα and PPARγ agonists, affected ApoB100 secretion. Huh7 cells were stimulated with 200 µM naringenin, 10 µM WY14,643, or 10 µM ciglitazone for 24 hours. Predictably, naringenin led to a 73%±9% (p<0.001) reduction in ApoB production (**Fig. 4a**) compared with a 33%±12% (p<0.01) reduction by the PPARα agonist WY14,643. Cigilitazone did not lead to a significant change in ApoB secretion.

Lastly, we characterized the metabolic changes induced by naringenin on primary hepatocytes. Primary rat hepatocytes were stimulated with 200 µM naringenin or 10 µM WY14,643 for 24 hours and culture media was analyzed for changes in urea, triglycerides, bile acid, and ketone bodies (**Fig. 4b**). As could be expected, primary hepatocytes showed no change in urea production. However, both naringenin and WY14,643 led to a 61% (p<0.001) and 41% (p<0.05) reduction in triglyceride production, respectively (**Fig. 4b**). Ketone body production was only slightly elevated by 17% and 23%, respectively. Importantly, no increase in intracellular levels of triglycerides were found (**Fig. 4c**) suggesting this inhibition was a result of increased fatty acid oxidation in primary hepatocytes. Interestingly, while PPARα agonist WY14,643 did not have an effect on hepatic bile acid production, naringenin cased a significant 32%±11% (p = 0.005) reduction in bile salt production (**Fig. 4b**), possibly due to inhibition of cholesterol synthesis, through suppression of LXRα [24], [25].

## Discussion

Dysregulation of lipid homeostasis is associated with multiple disease states, including metabolic, inflammatory, and infectious disorders [26]. Metabolic regulation is achieved in mammals through an intricate transcriptional mechanism responding to physiological cues. In recent years, a family of ligand-activated transcription factors called nuclear receptors emerged as key regulators of cellular metabolism [25], [27], [28]. Previously defined as *orphan receptors*, key metabolites were shown to be the natural ligands of many nuclear receptors, including liver X receptors (LXRs) which respond to oxysterols and glucose [29], [30], farnesoid X receptor (FXR) which responds to bile acids [31], and the peroxisome proliferator-activated receptors (PPARs) which respond to fatty acids [32].

The PPAR family includes PPARα, PPARγ, and PPARδ. The prevalence of these receptor subtypes varies in different tissues, with PPARα being the most prevalent subtype in the liver, and PPARγ the most abundant in adipose tissue [18]. PPARα is activated by fatty acids released in a physiological *fasting* state, leading to increased β-oxidation and gluconeogenesis [33], [34]. In clinical practice, PPARα agonists (fibrates) are used to treat hyperlipidemia, whereas PPARγ agonists (TZDs) are used to increase insulin sensitivity in muscle and adipose tissue [19], [35]. The LXR family includes both LXRα and LXRβ [29], [30], [36]. The latter is ubiquitously expressed, while the former is found primarily in the liver, adipose tissue, and macrophages and is activated by glucose and sterols [37], typical of a physiological *fed* state. In the liver, following activation by its ligands, LXRα activates lipogenic and glycolytic genes partly through activation of SREBP [38], [39]. HMGR, the target of statins, is regulated through this pathway controlling cholesterol availability for bile acid synthesis in hepatocytes.

Following a ligand binding event, both PPARs and LXRs become activated and heterodimerize with the retinoid X receptor (RXR) [27]. This heterodimer then binds conserved response elements such as PPRE or LXRE, while recruiting other co-regulatory molecules, such as the co-activators PGC1α [40] and Trap220 [41] for PPARα and LXRα, respectively. The requirement of a RXR binding partner leads to competitive inhibition at the level of receptor activation, offering a transcriptional layer of control over *fasted-to-fed* transition [42], [43], [44]. The existence of both a PPAR response element (PPRE) and an LXR response element (LXRE) in the regulatory region of LXRα [45], [46] suggests further levels of cross-regulation. Lastly, other coactivators, corepressors and kinases, such as PI3K and ERK, can regulate nuclear receptor activity by non-transcriptional mechanisms [47], [48], [49].

Naringenin is an aglycone of the grapefruit flavonoid naringin, which is responsible for the bitter taste in grapefruit. Naringenin has been reported to be an antioxidant with hypolipidemic, anti-carcinogenic and anti-inflammatory properties both *in vivo* and *in vitro* [5], [7], [8], [9], [10].The flavonoid was shown to reduce VLDL secretion [50], [51] through inhibition of ACAT2 and MTP [50], [52], critical enzymes for VLDL assembly. Allister *et al*. demonstrated that this inhibition is regulated through the MAPK/ERK pathway [52]. In addition, naringenin was shown to upregulate SREBP-dependent LDLR through PI3K activation [23]. Naringenin has also been shown to inhibit SREBP-dependent HMGR [53], while activating enzymes important in fatty acid oxidation such as CYP4A1 [54]. These myriad effects suggest that the flavonoid's target might be at the nuclear receptor level. Strengthening this hypothesis is the anecdotal report that naringenin binds to LXRα [23] and, more recently, that it induces PPRE activity in U-2OS cells [55].

In this work we demonstrate that naringenin activates the LBD of both PPARα and PPARγ using a reporter cell line over expressing GAL4 fusion proteins to either PPARα LBD or PPARγ LBD [20]. Activation of PPAR LBD releases the complex and allows it to bind the UAS_G_ response element, expressing luciferase. This reporter system demonstrates that naringenin acts on the LBD of both PPARα and PPARγ (**Fig. 1**), suggesting it serves as a natural ligand. However, the TR-FRET assay suggests that naringenin does not induce a conformational change in PPARα LBD like other ligands, such as GW7647, failing to increase its binding to the PGC1α co-activator (**Fig. 1**). One possibility is that naringenin induces a different conformational change in the PPARα LBD that recruits another co-activator, not found in the cell-free TR-FRET assay. However, a more likely scenario is that naringenin induces PPARα phosphorylation or alternately, PGC1α expression. Indeed our data shows that naringenin stimulation increases the mRNA abundance of PGC1α in Huh7 cells by 14-fold. Regardless of the exact nature of the interaction, naringenin-induced PPARα activation, lead to increased PPRE activity in human hepatocytes (**Fig. 3a**) and the expression of PPARα-regulated genes (**Fig. 3b**).

Concomitantly with PPARα activation, we show that naringenin inhibits the activity of LXRα. Using a similar reporter cell line over expressing the GAL4 fusion protein with LXRα LBD, we show a significant inhibition of LXRα LBD in the presence of TO901317, a classical agonist (**Fig. 2**). In contrast to the PPARα findings, we show that naringenin specifically increases the interaction of the Trap-220 co-activator with LXRα LBD in the cell-free TR-FRET assay. Interestingly, in the presence of LXRα agonist TO901317, naringenin actually decreased the interaction of Trap-220 with the LXRα LBD, demonstrating it is a partial agonist of LXRα naturally leading to a competitive inhibition of LXRα activity. This conclusion is further supported by the decrease in LXRE activity in human hepatocytes (**Fig 3c**) and the down-regulation of LXRα target genes (**Fig. 3d**).

The metabolic effect of PPARα induction and LXRα inhibition by naringenin are shown on gene expression (**Fig. 3**) and functional levels (**Fig. 4**). The mRNA abundance of PPARα-target genes that control fatty acid oxidation, such as CYP4A11, ACOX, and UCP1 significantly increases in human hepatoma cells. As lipid metabolism of hepatoma cell lines is dramatically lower than that of primary hepatocytes, we studied the metabolic aspects of PPARα and LXRα regulation in primary rat hepatocytes. As could be expected, both naringenin and PPARα agonist, WY14,643 led to a similar decrease in triglyceride production and an increase in ketone body secretion (**Fig. 4b**). Intracellular levels of hepatic triglycerides were also slightly reduced (**Fig. 4c**). Interestingly, naringenin caused a much steeper 73% decrease in VLDL secretion compared to 33% decrease by WY14,643 (**Fig. 4a**). This difference was significant (p = 0.006), and could possibly be due to inhibition of cholesterol synthesis through LXRα.

Indeed, the mRNA abundance of LXRα-target genes that regulates fatty acid and cholesterol synthesis, such as ABCA1, ABCG1, HMGR, and FASN decreses (**Fig. 3d**). While cholesterol production could not be detected in our system (*data not shown*), cholesterol serves as the percuror of hepatic bile acids. Interestingly, LXRα activation was shown to drive bile synthesis in rats [24], [25]. Therefore, the 32% decrease in bile acids prodcution following naringenin stimulation (**Fig. 4b**) serves as a surrogate measure of choelsterol production. WY14,643 which upregulates PPARα without effecting LXRα, showed no such change. Regretfully, no reliable LXRα inhibitor is commercially available, and 5CPPSS-50 showed significant toxicity in our hands (**Fig. S1**). Preliminary results using siRNA to LXRα show some inhibition of bile acid and VLDL production, although results were inconclusive (*data not shown*).

We note that the GAL4 fusion reporter data suggests that in-spite of the well known cross-regulation between PPARα and LXRα [42], [43], [44], naringenin appears to acts independently on each of these nuclear receptors. This is another indication of the nuclear receptor family promiscuity, and suggests that complex metabolic programs could be induced by relatively few compounds. Indeed, dual PPARα and PPARγ agonists have recently been investigated as normoglycemic and antiatherogenic agents [56]. Naringenin activation of both PPARα and PPARγ suggests a similar ability to regulate insulin sensitivity and LDL levels. However, in contrast to other dual PPAR agonists, such as Aleglitazar, our work shows naringenin is also an LXRα inhibitor. The metabolic program provoked by naringenin, appears to be a *fed-to-fasted* transition in the lipid metabolism of primary hepatocytes. Naringenin not only increases fatty acid oxidation but also inhibit fatty acid and cholesterol synthesis.

The potential of using a naturally occurring dietary supplement to regulate lipid metabolism is appealing as this by product of the grapefruit juice industry is non-toxic, cheap, and has demonstrated anti-inflammatory properties. This is especially important in the context of the rising costs of cardiovascular care, estimated by the AHA to rise above $500 billion this year. Naringenin ability to inhibit HMGR, the target of statins, while upregulating PPARα, the target of fibrates, suggest it can naturally find its place in the routine treatment of hyperlipidemia.

Finally, our group and other have shown that the Hepatitis C Virus (HCV) is critically dependent on host lipid metabolism [21], [57], [58]. Similar interplays were shown for the Hepatitis B Virus (HBV) [59], [60]. Therefore, compounds that modulate hepatic lipid metabolism could have significant antiviral effect. And indeed, our work shows that naringenin blocks HCV production from Huh7.5.1/JFH1 infected cells [21]. These findings form the basis of a currently conducted clinical trial to explore naringenin inhibition of HCV production in non-responding patients. Interestingly, the anti-inflammatory properties of naringenin could be readily explained in the context of PPAR activation. Such properties could have a significant effect on liver inflammation, preventing or delaying the development of hepatosteatosis and cancer [61].

## Materials and Methods

### Reagents

Fetal bovine serum (FBS), phosphate-buffered saline (PBS), Dulbecco's modified Eagle medium (DMEM), penicillin, streptomycin, trypsin-ethylene diamine tetraacetic acid (EDTA), OptiMEM basal medium, and Lipofectamine 2000 were obtained from Invitrogen Life Technologies (Carlsbad, CA). Insulin was obtained from Eli-Lilly (Indianapolis, IN). Dual luciferase assay kit was purchased from Promega (Madison, WI). The reported LXRα antagonist 5CPPSS-50 [62] was a kind gift of Dr. Hashimoto (The University of Tokyo). Unless otherwise noted, all other chemicals were purchased from Sigma-Aldrich Chemicals (St. Louis, MO).

### Cell culture

Huh7 cells were a kind gift of Prof. Raymong Chung, Massachusetts General Hospital. The cells were cultured in DMEM supplemented with 10% FBS, and 200 units/mL penicillin and streptomycin in a 5% CO_2_-humidified incubator at 37°C. Huh7 cells were passaged every 3 days and used at passage <15.

### GAL4-nuclear receptor activation assays

Activation of PPAR LBD was quantified using the previously described HGLN5 PPARα and PPARγ cell line [20]. Briefly, HeLa cells were stably transfected with the p(GAL4RE)5-βGlob-Luc-SVNeo plasmid, encoding the firefly luciferase gene driven by a pentamer of yeast activator GAL4 binding sites in front of β-globin promoter [20]. Cells were subsequently stably transfected with either pGAL4-PPARα-puro, or pGAL4-PPARγ-puro, encoding amino acids 1–147 of GAL4, followed by a short linker and the LBD of either PPARα or PPARγ, respectively [20]. HGLN5 cells were seeded at a density of 100,000 cells/cm^2^, test compounds were added 8 hours later and incubated for 16 hours. Following treatment, cells were washed with PBS and lysed in 25 mM Tris buffer (pH 7.8). Protein concentration was calculated using the Bradford assay and used to normalize the luciferase activity. Finally, activation of PPARα and PPARγ reporters is presented as percent of maximal activation by the known agonists GW7647 and BRL49653, respectively.

LXRα activation was investigated using the GeneBLAzer Beta-lactamase reporter technology (Invitrogen SelectScreen Cell-Based Nuclear Receptor Profiling Service, Madison, WI). LXR-alpha-UAS-bla HEK 293T cells were thawed and resuspended in Assay Media (DMEM phenol red free, 2% CD-treated FBS, 0.1 mM NEAA, 1 mM sodium pyruvate, 100 units/mL penicillin and streptomycin) to a concentration of 312,500 cells/mL. The control agonist TO901317 at the pre-determined EC_80_ concentration (5 nM) was added to wells containing variable concentrations of naringenin. The plate was incubated for 16–24 hours at 37°C and 5% CO_2_ in a humidified incubator. Substrate loading solution was added to each well and the plate is incubated for 2 hours at room temperature. The plate is read on a fluorescence plate reader. Results for each concentration (n = 4) are reported as percent activation of TO901317-stimulated, naringenin-free controls.

### TR-FRET Assays

LanthaScreen TR-FRET Coactivator Assays, purchased from Invitrogen (Madison, WI), were used to identify agonists and antagonists of PPARα and of LXRα. In these cell-free assays, ligands are identified by their ability to bind the recombinant LBD of the respective receptor and induce a conformational change that results in recruitment of a fluorescein-labeled coactivator peptide. A purified, glutathione S-transferase (GST)-tagged PPAR alpha or LXRα LBD is indirectly labeled using a terbium-labeled anti-GST tag antibody. Recruitment of fluorescein-labeled coactivator peptide – PGC1α for PPARα or Trap220 for LXRα – is measured by monitoring fluorescence resonance energy transfer (FRET) from the terbium-labeled antibody to the fluorescein on the peptide, resulting in a high TR-FRET ratio (520/490 nm emission). Test compounds were diluted in DMSO, and assays were run per the manufacturer's instructions. Briefly, to test the ability of a molecule to function as an agonist, increasing concentrations of naringenin or control agonist were added to LBD and co-activator peptide solutions. To test the ability of a molecule to function as an antgonist, a similar protocol was followed, but 250 nM TO901317 (EC80 of the agonist, measured in this assay) was added to all wells. In both agonist and antagonist modes, following 1 to 2 hour incubation at room temperature, the 520/490 TR-FRET ratio was measured with a PerkinElmer Envision fluorescent plate reader with TRF laser excitation using the following filter set: excitation 330 nm, emission 495 nm, and emission 520 nm. A 100 µsec delay followed by a 200 µsec integration time was used to collect the time-resolved signal. Results are displayed as percent activation compared to maximal activation of positive control.

### PPAR and LXRα response element luciferase reporter assays

Activation of PPRE and LXRE was quantified by transiently transfecting Huh7 cells with previously described firefly luciferase reporter plasmids, pACOX(×2)luc and pDR4(×2)luc, respectively [44], [63]. The pRL-TK plasmid (Promega, Madison, WI), constitutively expressing renilla luciferase, was co-transfected as positive control. pACOX(×2)luc was transfected into Huh7 cells cultured in OptiMEM. After 22 hours of culture, cells were stimulated with naringenin, WY14,643, or ciglitizone for 24 hours in standard culture medium. To quantify LXRE activity, cells were similarly transfected and treated with naringenin, 5CPPSS-50, or TO901317. Ratio of firefly to renilla luciferase luminescence was quantified using a Dual Luciferase Assay kit (Promega) following the manufacturer's instructions. DMSO levels were equal in all samples and never exceeded 0.5%. Results are reported as percent activation compared to DMSO-only controls.

### Quantitative Real Time Polymerase Chain Reaction (qRT-PCR)

Following a 24-hour stimulation, cells were lysed with RLT Plus buffer containing β-mercaptoethanol and RNA was isolated using RNeasy Mini Kit on a QIACube device (Qiagen, Valencia, CA). Total RNA was quantified on a ND-1000 spectrophotometer (NanoDrop Technologies, Rockland, Del.) and mRNA transcript abundance was measured on a MyiQ Real-Time PCR Detection System using iScript One-Step RT-PCR Kit With SYBR Green (Bio-Rad, Hercules, CA), according to the manufacturers' instructions. Primers used in these reactions (Integrated DNA Technologies, Coralville, IA) were designed using the PRIMER-BLAST program and appear in **Table 1**.

**Table 1:** Real-Time qRT-PCR Primers.

Gene	Primers
PPARα	ACG CTT TCA CCA GCT TCG AG
	GAA AGA AGC CCT TGC AGC CT
CYP4A11/22	ACT GGC TCT TCG GGC ACA TC
	ACA CGA ACT TTG CCT CCC CA
ACOX	TGG CAC ATA CGT GAA ACC GC
	CGC TGT ATC GGA TGG CAA TG
ApoAI	AAA GCT GCG GTG CTG ACC TT
	CGC TGT CTT TGA GCA CAT CCA
LXRα	GCT CCT TTT CTG ACC GGC TT
	TGA ATT CCA CTT GCA GCC CT
ABCA1	TCT GGA AAG CTC TGA AGC CG
	TGA GTT CCT CCC ACA TGC CT
ABCG1	ACC GGG GAA AAG TCT GCA AT
	TCA CCA GCC GAC TGT TCT GA
HMGR	GAC CCC TTT GCT TAG ATG AA
	GGA CTG GAA ACG GAT ATA AA
FASN	TTG CAG GGA GAC CTG GTG AT
	GGT GAG GGT GCT CAC AAA GG
PGC1α	GGC AGA AGA GCC GTC TCT ACT TA
	TTT GCA TGG TTC TGG GTA CTG A

### Human ApoB Enzyme-Linked Immunosorbent Assay (ELISA)

Huh7-secreted ApoB-100 was detected using ALerCHEK, Inc. (Portland, ME), total human ApoB-100 ELISA kit. The medium was diluted 1:10 with the specimen diluent, and the assay was carried out according to the manufacturer's directions.

### Analysis of metabolic changes in primary rat hepatocytes

Primary rat hepatocytes were harvested from adult female Lewis rats purchased from Charles River Laboratories, as previously described Hepatocyte viability was greater than 90% and purity above 95%. [64]. All animals were treated in accordance with National Research Council guidelines and approved by the Subcommittee on Research Animal Care at the Massachusetts General Hospital (IACUC #2005N000109). Cells were seeded on collagen-coated dishes at a density of 150,000 cells/cm^2^ under serum-free conditions, using 100 µL/mL soluble collagen type-I as attachment factor. Serum-free hepatocyte culture medium was purchased from Lonza (Walkersville, MD). Cells were stimulated with naringenin or WY14,643 for 24 hours, and cell culture medium was collected for metabolic analysis. Cell pellet was collected for intracellular triglyceride and total protein determination.

Urea concentration was measured using diacetylmonoxime methodology using a commercial available Blood Urea Nitrogen kit (Stanbio Labs, Boerne, TX). Triglycerides, in the culture medium and cell extracts, were quantified using a commercial kit (Sigma Chemical, St.Louis, MO) based on enzymatic hydrolysis by lipase to glycerol. Ketone bodies, were measured based on the appearance of NADH in conversion to acetoacetate in presence of b-hydroxybutyrate dehydrogenase (Zupke et al.1998). Total cholesterol was measured by a commercial available kit (StandBio Labs) based on the reaction of free cholesterol and cholesterol esters with cholesterol oxidase. Bile acids were determined through the formation of NADH in presence of the enzyme 3-α-hydroxysteroid dehydrogenase (Bio-Quant, San Diego, CA).

### Statistics

Data are expressed as the mean ± standard deviation. Statistical significance was determined by a one-tailed Student's t-test. A P-value of 0.05 was used for statistical significance.

## Supporting Information

Figure S1 5CPPSS-50 led to no change in LXRE activity. In all experiments, Renilla luciferase was used to account for variability in transfection efficiencies.
(3.87 MB TIF)

## References

[1] McGarry JD, Foster DW (1980). Regulation of hepatic fatty acid oxidation and ketone body production.. Annu Rev Biochem.

[2] Gastaldelli A, Kozakova M, Hojlund K, Flyvbjerg A, Favuzzi A (2009). Fatty liver is associated with insulin resistance, risk of coronary heart disease, and early atherosclerosis in a large European population.. Hepatology.

[3] Buchman A, Korenblatt K, Klein S, Schiff ER, Sorrell MF, Maddrey WC (2006). Nutrition and the Liver.. Schiff's diseases of the liver.

[4] Hogan P, Dall T, Nikolov P, Association AD (2003). Economic costs of diabetes in the US in 2002.. Diabetes Care.

[5] Wilcox LJ, Borradaile NM, Huff MW (1999). Antiatherogenic Properties of Naringenin, a Citrus Flavonoid.. Cardiovascular Drug Reviews.

[6] Crozier A, Jaganath IB, Clifford MN (2009). Dietary phenolics: chemistry, bioavailability and effects on health.. Natural product reports.

[7] Jung UJ, Kim HJ, Lee JS, Lee MK, Kim HO (2003). Naringin supplementation lowers plasma lipids and enhances erythrocyte antioxidant enzyme activities in hypercholesterolemic subjects.. Clinical Nutrition.

[8] Kurowska E, Borradaile N, Spence JD, Carroll KK (2000). Hypocholesterolemic effects of dietary citrus juices in rabbits.. Nutr Res.

[9] Lee C-H, Jeong T-S, Choi Y-K, Hyun B-H, Oh G-T (2001). Anti-atherogenic effect of citrus flavonoids, naringin and naringenin, associated with hepatic ACAT and aortic VCAM-1 and MCP-1 in high cholesterol-fed rabbits.. Biochem Biophys Res Commun.

[10] Kim S-Y, Kim H-J, Lee M-K, Jeon S-M, Do G-M (2006). Naringin time-dependently lowers hepatic cholesterol biosynthesis and plasma cholesterol in rats fed high-fat and high-cholesterol diet.. J Med Food.

[11] Wilcox LJ, Borradaile NM, Dreu LEd, Huff MW (2001). Secretion of hepatocyte apoB is inhibited by the flavonoids, naringenin and hesperetin, via reduced activity and expression of ACAT2 and MTP.. Journal of Lipid Research.

[12] Kurowska EM, Manthey JA, Casaschi A, Theriault AG (2004). Modulation of HepG2 Cell Net Apolipoprotein B Secretion by the Citrus Polymethoxyflavone, Tangeretin.. Lipids.

[13] Borradaile NM, Dreu LEd, Barrett PHR, Huff MW (2002). Inhibition of hepatocyte apoB secretion by naringenin: enhanced rapid intracellular degradation independent of reduced microsomal cholesteryl esters.. Journal of Lipid Research.

[14] Borradaile NM, Dreu LEd, Huff MW (2003). Inhibition of Net HepG2 Cell Apolipoprotein B Secretion by the Citrus Flavonoid Naringenin Involves Activation of Phosphatidylinositol 3-Kinase, Independent of Insulin Receptor Substrate-1 Phosphorylation.. Diabetes.

[15] Huong DT, Takahashi Y, Ide T (2006). Activity and mRNA levels of enzymes involved in hepatic fatty acid oxidation in mice fed citrus flavonoids.. Nutrition.

[16] Liu L, Shan S, Zhang K, Ning Z-Q, Lu X-P (2008). Naringenin and Hesperetin, Two Flavonoids Derived from Citrus aurantium Up-regulate Transcription of Adiponectin.. Phytotherapy Research.

[17] Mulvihill EE, Allister EM, Sutherland BG, Telford DE, Sawyez CG (2009). Naringenin prevents dyslipidemia, apoB overproduction and hyperinsulinemia in LDL-receptor null mice with diet-induced insulin resistance.. Diabetes.

[18] Lee C-H, Olson P, Evans RM (2003). Minireview: lipid metabolism, metabolic diseases, and peroxisome proliferator-activated receptors.. Endocrinology.

[19] Gervois P, Fruchart J, Staels B (2007). Drug Insight: mechanisms of action and therapeutic applications for agonists of peroxisome proliferator-activated receptors.. Nature clinical practice Endocrinology & metabolism.

[20] Seimandi M, Lemaire G, Pillon A, Perrin A, Carlavan I (2005). Differential responses of PPARalpha, PPARdelta, and PPARgamma reporter cell lines to selective PPAR synthetic ligands.. Anal Biochem.

[21] Nahmias Y, Goldwasser J, Casali M, van Poll D, Wakita T (2008). Apolipoprotein B-dependent hepatitis C virus secretion is inhibited by the grapefruit flavonoid naringenin.. Hepatology.

[22] Lee SH, Park YB, Bae KH, Bok SH, Kwon YK (1999). Cholesterol-lowering activity of naringenin via inhibition of 3-hydroxy-3-methylglutaryl coenzyme A reductase and acyl coenzyme A:cholesterol acyltransferase in rats.. Ann Nutr Metab.

[23] Borradaile NM, de Dreu LE, Huff MW (2003). Inhibition of net HepG2 cell apolipoprotein B secretion by the citrus flavonoid naringenin involves activation of phosphatidylinositol 3-kinase, independent of insulin receptor substrate-1 phosphorylation.. Diabetes.

[24] Gupta S, Pandak WM, Hylemon PB (2002). LXR alpha is the dominant regulator of CYP7A1 transcription.. Biochem Biophys Res Commun.

[25] di Fabiani E, Crestani M, Mitro N (2004). Lipid-activated nuclear receptors: from gene transcription to the control of cellular metabolism.. Eur J Lipid Sci Technol.

[26] Mittra S, Bansal VS, Bhatnagar PK (2008). From a glucocentric to a lipocentric approach towards metabolic syndrome.. Drug Discovery Today.

[27] Gronemeyer H, Gustafsson JA, Laudet V (2004). Principles for modulation of the nuclear receptor superfamily.. Nat Rev Drug Discov.

[28] Ory DS (2004). Nuclear receptor signaling in the control of cholesterol homeostasis: have the orphans found a home?. Circulation Research.

[29] Mitro N, Mak PA, Vargas L, Godio C, Hampton E (2007). The nuclear receptor LXR is a glucose sensor.. Nature.

[30] Edwards PA, Kennedy MA, Mak PA (2002). LXRs; oxysterol-activated nuclear receptors that regulate genes controlling lipid homeostasis.. Vascul Pharmacol.

[31] Sinal CJ, Tohkin M, Miyata M, Ward JM, Lambert G (2000). Targeted disruption of the nuclear receptor FXR/BAR impairs bile acid and lipid homeostasis.. Cell.

[32] Gottlicher M, Widmark E, Li Q, Gustafsson JA (1992). Fatty acids activate a chimera of the clofibric acid-activated receptor and the glucocorticoid receptor.. Proc Natl Acad Sci U S A.

[33] Martin PGP, Guillou H, Lasserre F, Déjean S, Lan A (2007). Novel aspects of PPARalpha-mediated regulation of lipid and xenobiotic metabolism revealed through a nutrigenomic study.. Hepatology.

[34] Van Raalte DH, Min L, Pritchard PH, Wasan KM (2004). Peroxisome proliferator-activated receptor (PPAR)-alpha: a pharmacological target with a promising future.. Pharm Res.

[35] Vuppalanchi R, Chalasani N (2009). Nonalcoholic fatty liver disease and nonalcoholic steatohepatitis: Selected practical issues in their evaluation and management.. Hepatology.

[36] Millatt LJ, Bocher V, Fruchart J-C, Staels B (2003). Liver X receptors and the control of cholesterol homeostasis: potential therapeutic targets for the treatment of atherosclerosis.. Biochim Biophys Acta.

[37] Forman, Ruan, Chen, Schroepfer, Evans (1997). The orphan nuclear receptor LXRalpha is positively and negatively regulated by distinct products of mevalonate metabolism.. Proc Natl Acad Sci USA.

[38] Yoshikawa T, Shimano H, Amemiya-Kudo M, Yahagi N, Hasty A (2001). Identification of liver X receptor-retinoid X receptor as an activator of the sterol regulatory element-binding protein 1c gene promoter.. Molecular and Cellular Biology.

[39] Pawar, Botolin, Mangelsdorf, Jump (2003). The Role of Liver X Receptor-α in the Fatty Acid Regulation of Hepatic Gene Expression.. Journal of Biological Chemistry.

[40] Vega RB, Huss JM, Kelly DP (2000). The coactivator PGC-1 cooperates with peroxisome proliferator-activated receptor alpha in transcriptional control of nuclear genes encoding mitochondrial fatty acid oxidation enzymes.. Molecular and Cellular Biology.

[41] Son YL, Lee YC (2009). Molecular determinants of the interactions between LXR/RXR heterodimers and TRAP220.. Biochem Biophys Res Commun.

[42] Ide T, Shimano H, Yoshikawa T, Yahagi N, Amemiya-Kudo M (2003). Cross-talk between peroxisome proliferator-activated receptor (PPAR) alpha and liver X receptor (LXR) in nutritional regulation of fatty acid metabolism. II. LXRs suppress lipid degradation gene promoters through inhibition of PPAR signaling.. Molecular Endocrinology.

[43] Yoshikawa T, Ide T, Shimano H, Yahagi N, Amemiya-Kudo M (2003). Cross-talk between peroxisome proliferator-activated receptor (PPAR) alpha and liver X receptor (LXR) in nutritional regulation of fatty acid metabolism. I. PPARs suppress sterol regulatory element binding protein-1c promoter through inhibition of LXR signaling.. Molecular Endocrinology.

[44] Miyata KS, McCaw SE, Patel HV, Rachubinski RA, Capone JP (1996). The orphan nuclear hormone receptor LXR alpha interacts with the peroxisome proliferator-activated receptor and inhibits peroxisome proliferator signaling.. J Biol Chem.

[45] Laffitte BA, Joseph SB, Walczak R, Pei L, Wilpitz DC (2001). Autoregulation of the human liver X receptor alpha promoter.. Molecular and Cellular Biology.

[46] Tobin KA, Steineger HH, Alberti S, Spydevold O, Auwerx J (2000). Cross-talk between fatty acid and cholesterol metabolism mediated by liver X receptor-alpha.. Molecular Endocrinology.

[47] Hu X, Li S, Wu J, Xia C, Lala D (2003). Liver X receptors interact with corepressors to regulate gene expression.. Molecular Endocrinology.

[48] Rochette-Egly C (2003). Nuclear receptors: integration of multiple signalling pathways through phosphorylation.. Cellular Signal.

[49] McKenna NJ, O'Malley BW (2002). Combinatorial Control of Gene Expression by Nuclear Receptors and Coregulators.. Cell.

[50] Wilcox LJ, Borradaile NM, de Dreu LE, Huff MW (2001). Secretion of hepatocyte apoB is inhibited by the flavonoids, naringenin and hesperetin, via reduced activity and expression of ACAT2 and MTP.. J Lipid Res.

[51] Borradaile NM, de Dreu LE, Barrett PH, Huff MW (2002). Inhibition of hepatocyte apoB secretion by naringenin: enhanced rapid intracellular degradation independent of reduced microsomal cholesteryl esters.. J Lipid Res.

[52] Allister EM, Borradaile NM, Edwards JY, Huff MW (2005). Inhibition of microsomal triglyceride transfer protein expression and apolipoprotein B100 secretion by the citrus flavonoid naringenin and by insulin involves activation of the mitogen-activated protein kinase pathway in hepatocytes.. Diabetes.

[53] Jeon SM, Kim HK, Kim HJ, Do GM, Jeong TS (2007). Hypocholesterolemic and antioxidative effects of naringenin and its two metabolites in high-cholesterol fed rats.. Transl Res.

[54] Huong DT, Takahashi Y, Ide T (2006). Activity and mRNA levels of enzymes involved in hepatic fatty acid oxidation in mice fed citrus flavonoids.. Nutrition (Burbank, Los Angeles County, Calif).

[55] Liu L, Shan S, Zhang K, Ning Z-Q, Lu X-P (2008). Naringenin and hesperetin, two flavonoids derived from Citrus aurantium up-regulate transcription of adiponectin.. Phytotherapy research: PTR.

[56] Chang F, Jaber LA, Berlie HD, O'Connell MB (2007). Evolution of peroxisome proliferator-activated receptor agonists.. Ann Pharmacother.

[57] Gastaminza P, Cheng G, Wieland S, Zhong J, Liao W (2008). Cellular determinants of hepatitis C virus assembly, maturation, degradation, and secretion.. Journal of Virology.

[58] Huang H, Sun F, Owen DM, Li W, Chen Y (2007). Hepatitis C virus production by human hepatocytes dependent on assembly and secretion of very low-density lipoproteins.. Proc Natl Acad Sci USA.

[59] Kim K, Kim, Kim KH, Cheong J (2008). Hepatitis B virus X protein induces lipogenic transcription factor SREBP1 and fatty acid synthase through the activation of nuclear receptor LXRalpha.. Biochem J.

[60] Na T-A, Shin YK, Roh KJ, Kang SA, Hong I (2008). Liver X receptor mediates hepatitis B virus X protein-induced lipogenesis in hepatitis B virus-associated hepatocellular carcinoma.. Hepatology.

[61] Mahmood S, Togawa K, Kawanaka M, Niiyama G, Yamada G (2008). An Analysis of Risk Factors for Developing Hepatocellular Carcinoma in a Group of Hepatitis C Patients with Stage 3 Fibrosis following Interferon Therapy.. Cancer Inform.

[62] Noguchi-Yachide T, Miyachi H, Aoyama H, Aoyama A, Makishima (2007). Structural development of liver X receptor (LXR) antagonists derived from thalidomide-related glucosidase inhibitors.. Chem Pharm Bull.

[63] Marcus SL, Miyata KS, Zhang B, Subramani S, Rachubinski RA (1993). Diverse peroxisome proliferator-activated receptors bind to the peroxisome proliferator-responsive elements of the rat hydratase/dehydrogenase and fatty acyl-CoA oxidase genes but differentially induce expression.. Proc Natl Acad Sci USA.

[64] Kidambi S, Yarmush RS, Novik E, Chao P, Yarmush ML (2009). Oxygen-mediated enhancement of primary hepatocyte metabolism, functional polarization, gene expression, and drug clearance.. Proc Natl Acad Sci U S A.

