# Supplementary figures and images for: Transcriptional Regulation of Human and Rat Hepatic Lipid Metabolism by the Grapefruit Flavonoid Naringenin: Role of PPARα, PPARγ and LXRα
Source: PLoS One. 2010 Aug 25;5(8):e12399. doi: 10.1371/journal.pone.0012399 (PMC2928300; doi:10.1371/journal.pone.0012399)

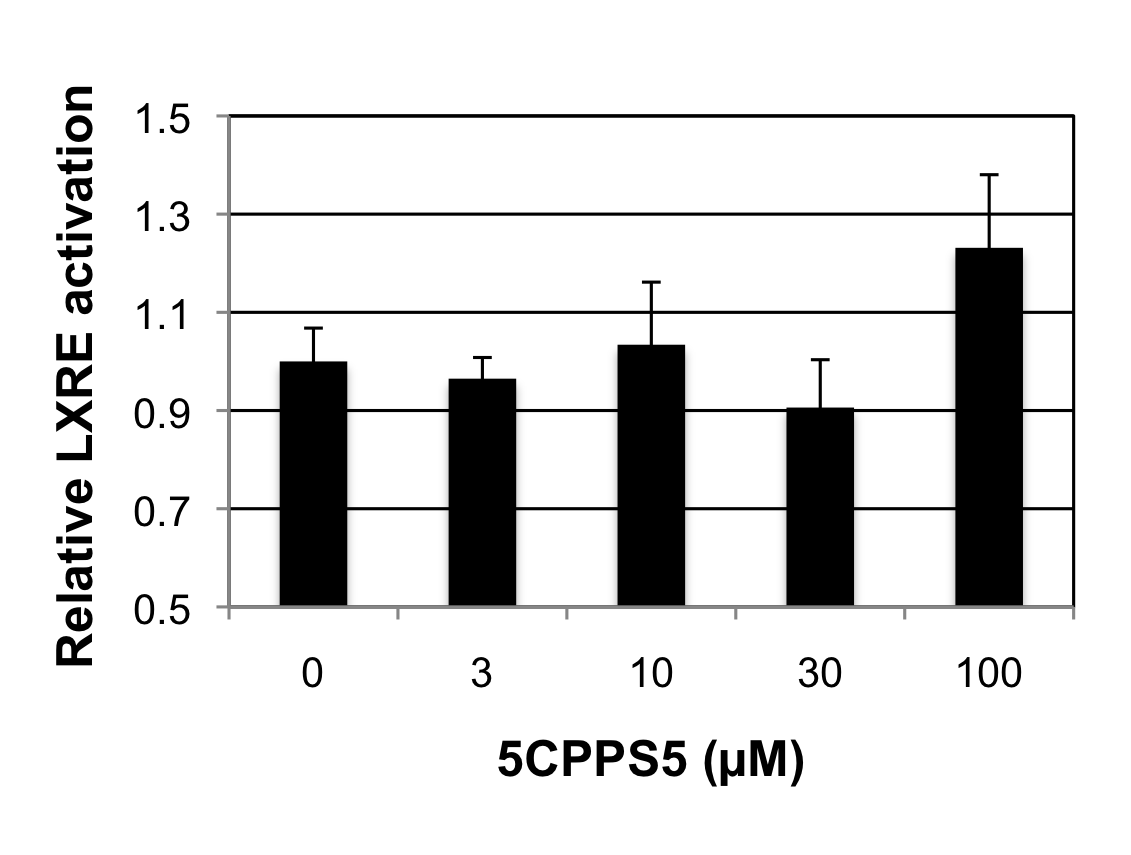

Supplement: Figure S1 — 5CPPSS-50 led to no change in LXRE activity. In all experiments, Renilla luciferase was used to account for variability in transfection efficiencies. (3.87 MB TIF) [file pone.0012399.s001.tif]
